# Density-Dependent Benefits in Ant-Hemipteran Mutualism? The Case of the Ghost Ant *Tapinoma melanocephalum* (Hymenoptera: Formicidae) and the Invasive Mealybug *Phenacoccus solenopsis* (Hemiptera: Pseudococcidae)

**DOI:** 10.1371/journal.pone.0123885

**Published:** 2015-04-17

**Authors:** Aiming Zhou, Beiqing Kuang, Yingrui Gao, Guangwen Liang

**Affiliations:** 1 Hubei Insect Resources Utilization and Sustainable Pest Management Key Laboratory, College of Plant Science and Technology, Huazhong Agricultural University, Wuhan, People’s Republic of China; 2 Red Imported Fire Ant Research Center, South China Agricultural University, Guangzhou 510642, China; Arizona State University, UNITED STATES

## Abstract

Although density-dependent benefits to hemipterans from ant tending have been measured many times, few studies have focused on integrated effects such as interactions between ant tending, natural enemy density, and hemipteran density. In this study, we tested whether the invasive mealybug *Phenacoccus solenopsis* is affected by tending by ghost ants (*Tapinoma melanocephalum*), the presence of parasitoids, mealybug density, parasitoid density and interactions among these factors. Our results showed that mealybug colony growth rate and percentage parasitism were significantly affected by ant tending, parasitoid presence, and initial mealybug density separately. However, there were no interactions among the independent factors. There were also no significant interactions between ant tending and parasitoid density on either mealybug colony growth rate or percentage parasitism. Mealybug colony growth rate showed a negative linear relationship with initial mealybug density but a positive linear relationship with the level of ant tending. These results suggest that benefits to mealybugs are density-independent and are affected by ant tending level.

## Introduction

The relationship between hemipterans and ants is generally thought to be mutualistic because both partners appear to benefit from an association [1]. Interactions between ants and hemipterans have been widely recognized and documented [2–5]. Ant tending improves the survival and the reproduction of aphid colonies, primarily by protecting the hemipterans from attack by natural enemies. In exchange for tending the hemipterans, ants receive large amounts of honeydew in a consumer-resource mutualism [2, 6]. Mutualisms can persist surprisingly well when the trade-offs between the cost and the benefit are in balance [7]. Many studies have shown that the stability of mutualisms can be affected by numerous factors, such as the density of the ants and hemipterans [8–10], host quality [11–12] and populations of hemipteran enemies [13–14]. The costs and benefits between the mutual partners vary greatly in both space and time, inevitably causing outcomes within most interactions to vary as well [15].

Benefits from mutualisms are usually dependent upon variations in the environment and the density of the interacting species [16]. For example, interactions between *Heliconius* butterflies and their Mullerian mimics benefit one another at a low density, but this advantage is lost at higher density [17]. Ant tending significantly increases the growth of low-density aphid populations, but the positive effect of ant tending decreases as aphid density increases [8]. Although a few studies of ant-hemipteran mutualisms have reported the patterns of density-dependent benefits to hemipterans [8–9, 18], most of the studies focused just on the individual factors affecting the benefits to hemipterans. For example, Morales (2000) documented that benefits to the treehopper *Publilia concava* depends on the density of treehoppers [10]. Similar studies have also demonstrated density-dependent benefits in different mutualistic systems, such as the interaction between *Aphis varians* and *Formica cinerea* [8] or *Publilia modesta* and *Formica altipetens* [9]. However, the services to hemipterans provided by ant tending can vary concurrently with changes in multiple factors. Heretofore, no studies have experimentally addressed the role of ant tending, hemipteran density, enemy density, and their interactions in generating patterns of mutualism.

The mealybug *Phenacoccus solenopsis* Tinsley (Hemiptera: Pseudococcidae) is native to the United States [19] but caused serious damage to cotton in India and Pakistan in 2005 [20]. Currently, the mealybug is an important invasive species in China [21]. The ghost ant *Tapinoma melanocephalum* is a worldwide invasive species for which the native range is unknown but is believed to be Africa or Asia [22]. The ghost ant is highly adaptable in its nesting habits and has been in China for a long time. These ants are fond of honeydew and tend honeydew-excreting insects [23]. Our previous studies have shown that ghost ants have established a close relationship with *P*. *solenopsis* in China, and the persistence of the mutualistic relationship under low mealybug density is greater than that under high density [5]. The parasitoid *Aenasius bambawalei* is an important enemy of *P*. *solenopsis* in China [24]. *A*. *bambawalei* has also been reported as a solitary endoparasitoid of *P*. *solenopsis* [25–27]. It is also the most dominant and aggressive parasitoid reported thus far [28–29]. The performance of *A*. *bambawalei* was significantly reduced by ghost ant tending [30]. Those results may suggest that mutualism between *T*. *melanocephalum* and *P*. *solenopsis* is conditional and affected by multiple factors. While this form of conditional mutualism has received less attention, there are a few examples in the literature.

In this study, we conducted a series of experiments to test the effects of ant tending, mealybug density, parasitoid density, and their interactions on the benefits to the mealybug. Furthermore, we examined the relationship between the level of ant tending and the benefits to the mealybug.

## Materials and Methods

### Plants and Insects

Cotton plants were cultivated in plastic flowerpots (18 cm × 14 cm × 17 cm) in a greenhouse. Each plant was approximately 25 cm in height and had 20 true leaves. Colonies of *P*. *solenopsis* were collected from the campus of South China Agricultural University and placed on the cotton plants. The 1^st^ instar mealybug nymphs were inoculated on the cotton and raised for several generations. The mealybug colonies were reared in the laboratory with the temperature maintained at 27±2°C and a relative humidity of 60–70%. Colonies of ghost ants were collected from experimental fields of South China Agricultural University in the suburbs of Guangzhou (113°37′56”E, 23°14′22′′N). The colonies were separated from the soil by dripping water into plastic boxes containing soil and ants until the ant colonies floated [31]. One subcolony (approximately 1.0 g) from each colony was prepared using a microbalance (Sartorius, BS, 224S). Each subcolony included one queen, adult workers (approximately 1500 individuals), pupae, larvae, and eggs. The ants were placed in a 9-cm plastic petri dish, which served as an artificial nest. The *T*. *melanocephalum* subcolonies were maintained with distilled water plus a 10% honey solution, which was distributed through tubes. An enemy of *P*. *solenopsis*, the parasitoid *Aenasius bambawalei* was also collected from *Hibiscus rosa-sinensis* in the experimental field. *A*. *bambawalei* were collected as mummified mealybugs, which were separated into gel capsules (10 mm in length) until adult emergence. Then, the wasps were randomly paired and allowed to copulate. Copulation was observed in all pairings, and the fertilized female wasps were used in the experiments 24 h after the initial pairing.

## Experimental Design

### Experiment 1: Effects of ant tending, parasitoids, and variation in mealybug density on the benefits to mealybugs

We measured the effects of ant tending (present/absent), parasitoid (present/absent) and the initial mealybug density (low/medium/high) on mealybug colony growth. This experiment used a full-factorial 3-way design. There were twelve combinations of ants (present/absent) × densities (low/medium/high) × parasitoids (present/absent). Each combination was repeated ten times. We grew the cotton (20 true leaves and approximately 25 cm tall) in plastic boxes filled with soil. Each plastic box was surrounded by a cage (70 cm × 70 cm × 100 cm) covered with nylon netting. A subcolony of *T*. *melanocephalum* was placed in each plastic box, and the ants constructed new nests in the soil immediately. The ants were given two mealworms and water (50 mL) every two days. A different number of 2^nd^ instar mealybugs were transferred onto the cotton plants. Mealybug density on each plant was classified as low density (10 individuals per plant), medium density (50 individuals per plant), and high density (100 individuals per plant). The mealybug larvae were transferred onto the plants through small plastic tubes with cotton plugs. When the plug was removed, mealybugs crawled out from the tubes and began sucking the tender plant leaves on the top branches of each cotton plant. After 24 h, two fertilized female parasitoids were placed on each selected caged plant. In our study, the parasitic pressure is defined as the number of parasitoids per plant, so the parasitic pressure is the same among the treatments. The design was consistent with the study by Itioka and Inoue [13], and the experiments lasted for 8 weeks. All surviving mealybugs and mummified mealybugs on each plant were collected and counted. The effects of ant tending and mealybug density on the colony growth rate of mealybugs were analyzed. We defined the colony growth rate of mealybugs as the final population density divided by the initial population density. The parasitism rate was defined as the number of mummified mealybugs divided by the total number of mealybugs (mummified and surviving mealybugs) on each plant. The mummified mealybugs are easily identified by the decreasing wax and the body color of the mealybugs.

### Experiment 2: Effects of ant tending and variation in parasitoid density on the benefits to mealybugs

In this experiment, we tested whether mealybug colony growth was affected by ant tending and variation in parasitoid density. The procedures used in this experiment were similar to those used in experiment 1. Thirty 2^nd^ instar mealybugs were transferred onto each cotton plant (30 individuals per plant). After 24 h, fertilized female parasitoids were placed on each caged plant. We assigned a different density of parasitoid to indicate different levels of parasitic pressure. Parasitoid density on each plant was classified as low density (1 individual per plant) or high density (4 individuals per plant). The treatments were as follows: 1) ant tending at low parasitic pressure; 2) ant tending at high parasitic pressure; 3) low parasitic pressure without ant tending; and 4) high parasitic pressure without ant tending. All treatments were repeated 10 times. After 8 weeks, we counted and recorded the surviving mealybugs and mummified mealybugs on each plant.

### Experiment 3: Relationship between the benefits from ant tending and the level of ant tending

In this experiment, we examined whether the level of ant tending was influenced by the mealybug density under parasitic pressure. We defined the ant tending level as the mean number of tending ants per mealybug. On a gradient with a total of twenty densities, the initial population density ranged from 10 to 200 mealybugs per plant. Each density included two treatments: 1) mealybugs with parasitoids and with ant tending and 2) mealybugs with parasitoids and without ant tending. In our experiments, the parasitic pressure is defined as the number of parasitoids per plant, so the parasitic pressure is the same between treatments. Two fertilized female parasitoids were placed on each caged plant in this experiment. The experiment lasted for 8 weeks, and every two weeks we counted the number of surviving and mummified mealybugs and the number of tending ants on the plant.

## Statistical Analyses

To satisfy the preconditions of the analysis of variance, the data were transformed. The growth rate of the mealybug colony was log-transformed, and the percentage of parasitism on the mealybugs was treated by the arcsine square root-transformation. When the data were normally distributed and had similar variances, an analysis of variance (ANOVA) using the Type III sum of squares was performed to compare the means among all measured variables. A linear regression model was performed to analyze the relationship between benefits from ant tending and the ant tending level. Analysis of covariance was used to test the difference in the slopes of the linear model. All statistical analyses were conducted using SPSS version 14.0 (SPSS Inc., Chicago, IL).

## Results

### The effects of ant tending, parasitoids, and mealybug density on mealybug colony growth and parasitism

**Fig 1:**
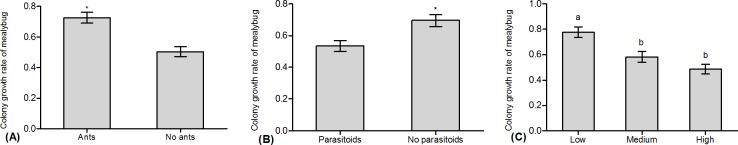
Effect of ant tending, the presence of parasitoids, and initial mealybug density on mealybug colony growth. (A): Ant tending; (B): Parasitoid; (C): Initial mealybug density.

**Fig 2:**
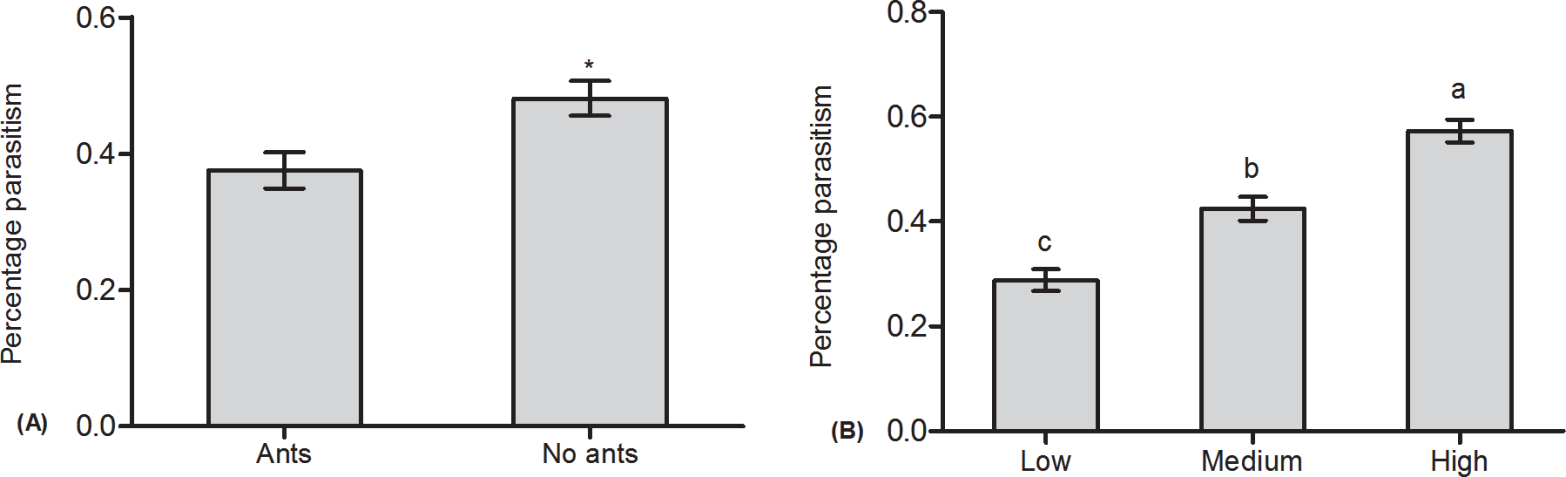
Effect of ant tending, the presence of parasitoids, and initial mealybug density on the percentage of parasitism. (A): Ant tending; (B): Initial mealybug density.

Our results showed that the growth rate of the mealybug colony was significantly affected by ant tending, parasitoids, and the initial mealybug density, separately (Table 1: Ant tending, Parasitoid, Density). The growth rate was obviously improved by ant tending (Table 1: Ant tending, Fig 1A, and S1 Dataset). In contrast, it showed a notable decrease with the presence of the parasitoid or with a higher initial mealybug density (Table 1: Parasitoid, Fig 1B; Table 1: Density, Fig 1C; and S1 Dataset). No significant interactions were found for ant tending and parasitoids, ant tending and mealybug density, and parasitoids and mealybug density, nor for all three factors together (Table 1). The effect of ant tending and initial mealybug density significantly affected the percentage of parasitism (Table 2: Ant tending, Density). The percentage of parasitism of mealybugs significantly decreased with ant tending but increased with increased mealybug density (Table 2: Ant tending, Fig 2A; Table 2: Density, Fig 2B; and S1 Dataset). However, the effect of interactions between ant tending and mealybug density on percentage of parasitism was indistinct (Table 2: Ant tending × Density).

**Table 1:** Analysis of variance of the mealybug colony growth rate with ant tending, parasitoid, and initial mealybug density.

Source of variation	SS	df	MS	*F*	*P*
Ant tending	1.478	1	1.478	28.092	<0.001
Parasitoid	0.775	1	0.775	14.729	<0.001
Density	1.754	2	0.877	16.669	<0.001
Ant tending × Parasitoid	0.016	1	0.016	0.311	0.578
Ant tending × Density	0.060	2	0.030	0.566	0.569
Parasitoid × Density	0.080	2	0.040	0.761	0.470
Ant tending × Parasitoid × Density	0.015	2	0.007	0.138	0.871
Error	5.682	108	0.053		

**Table 2:** Analysis of variance of the percentage parasitism with ant tending and initial mealybug density.

Source of variation	SS	df	MS	*F*	*P*
Ant tending	0.165	1	0.165	23.583	<0.001
Density	0.810	2	0.405	57.731	<0.001
Ant tending × Density	0.005	2	0.002	0.328	0.722
Error	0.379	54	0.007		

### The effect of ant tending and parasitoid density on mealybug colony growth and parasitism

**Fig 3:**
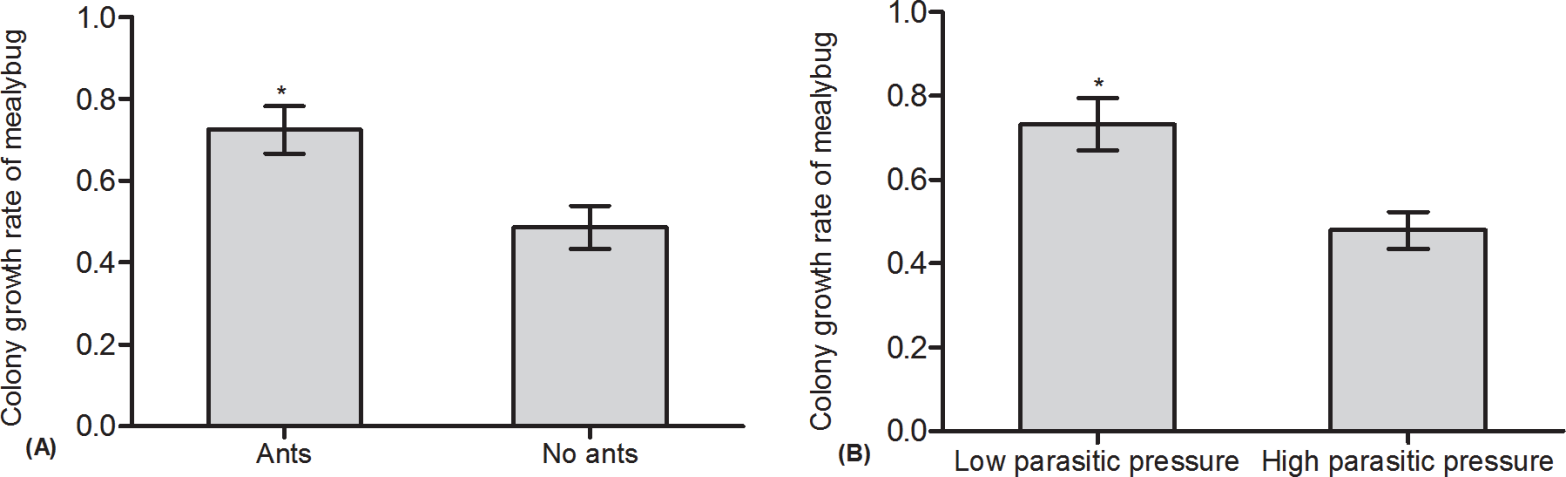
Effect of ant tending and parasitoid density on mealybug colony growth. (A): Ant tending; (B): Parasitoid density.

**Fig 4:**
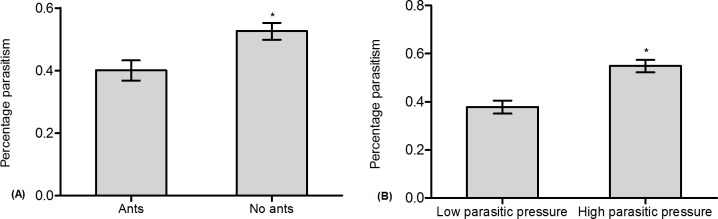
Effect of ant tending and parasitoid density on the percentage of parasitism. (A): Ant tending; (B): Parasitoid density.

The results showed that the effects of ant tending and parasitoid density on the colony growth rate of mealybugs were significant (Table 3: Ant tending, Parasitoid density). Specifically, the colony growth rate with ant tending was obviously greater than without ant tending (Table 3: Ant tending, Fig 3A, and S2 Dataset). The colony growth rate under low parasitic pressure was significantly greater than that under high parasitic pressure (Table 3: Parasitoid density, Fig 3B, and S2 Dataset). No significant effects on the colony growth rate of mealybugs were found for the interactions between ant tending and parasitoid density (Table 3: Ant tending × Parasitoid density). In addition, ant tending and parasitoid density also significantly affected the percentage of parasitism (Table 4: Ant tending, Parasitoid density). The percentage of parasitism without ant tending was obviously greater than that with ant tending (Table 4: Ant tending, Fig 4A, and S2 Dataset). The percentage of parasitism under high parasitic pressure was significantly greater than that under low parasitic pressure (Table 4: Parasitoid density, Fig 4B, and S2 Dataset). However, the effect of the interactions between ant tending and parasitoid density on the percentage of parasitism was not significant (Table 4: Ant tending × Parasitoid density).

**Table 3:** Analysis of variance of the mealybug colony growth rate with ant tending and parasitic pressure.

Source of variation	SS	df	MS	*F*	*P*
Ant tending	0.569	1	0.569	12.410	0.001
Parasitoid density	0.638	1	0.638	13.910	0.001
Ant tending × Parasitoid density	0.044	1	0.044	0.950	0.336
Error	1.650	36	0.046		

**Table 4:** Analysis of variance of the percentage parasitism with ant tending and parasitic pressure.

Source of variation	SS	df	MS	*F*	*P*
Ant tending	0.157	1	0.157	14.874	<0.001
Parasitoid density	0.291	1	0.291	27.482	<0.001
Ant tending × Parasitoid density	0.005	1	0.005	0.462	0.501
Error	0.381	36	0.011		

### The relationship between benefits from ant tending and ant tending level

**Fig 5:**
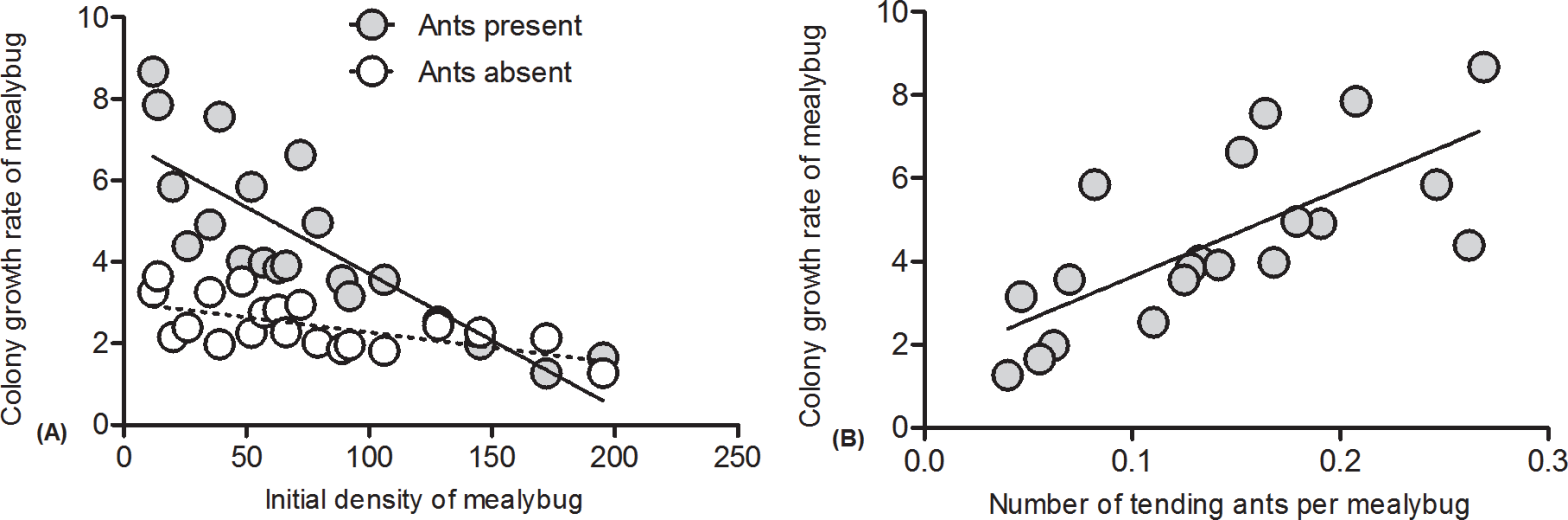
Relationship between mealybug colony growth and level of ant tending. (A): Initial mealybug density and growth rate of the mealybugs; (B): Level of ant tending and growth rate of mealybugs.

**Fig 6:**
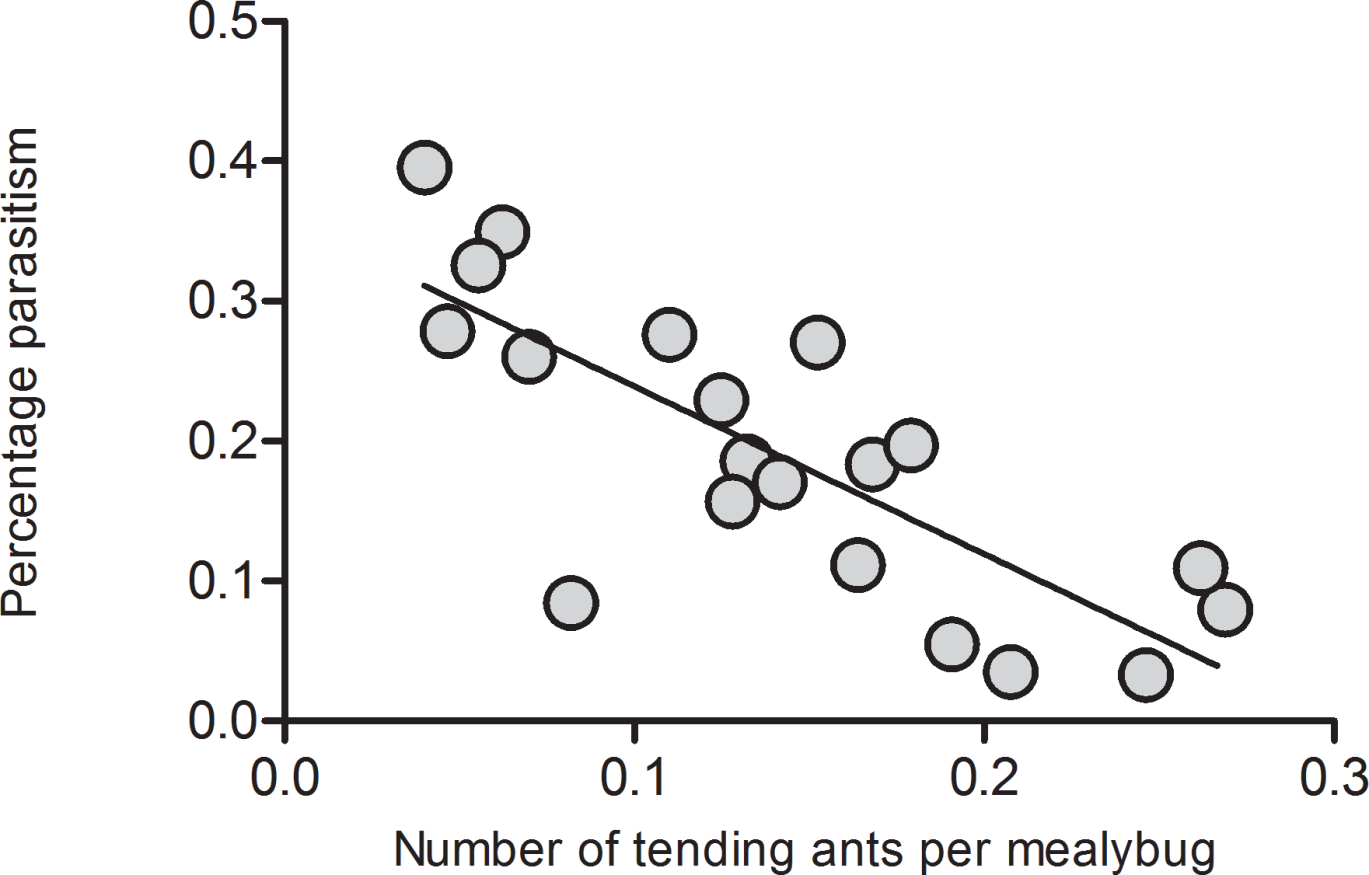
Relationship between percentage of parasitism and ant tending level. * above bars indicates statistically significant differences between the two treatments ( P<0.05), different letters above bars indicate significant differences among the treatments (P<0.05).

The colony growth rate of mealybugs showed a negative linear relationship with the initial mealybug density both in the presence and in the absence of ants (Fig 5A, Ants present: Y = -0.033X+6.979, R^2^ = 0.679, *P*<0.001; Ants absent: Y = -0.007X+3.009, R^2^ = 0.377, *P* = 0.004; and S3 Dataset), and there was a significant difference in the slopes of those two lines (F = 19.323, *df* = 1, *P*<0.001). When the mealybug density was greater than approximately 150 individuals per plant, the growth rate of the mealybug colony on ant-tended plants was less than that on untended plants (Fig 5A). In contrast, there was a positive linear relationship between the level of ant tending and the colony growth rate of mealybugs (Fig 5B: Y = 20.911X+1.545, R^2^ = 0.515, *P*<0.001, and S3 Dataset). There was a negative linear relationship between the percentage of parasitism and the level of ant tending (Fig 6: Y = -1.198X+0.359, R^2^ = 0.619, *P*<0.001, and S3 Dataset).

## Discussion

The benefits of ant tending to hemipterans are widely recognized. Although several studies have analyzed the relationship between the benefits from mutualism and the density of hemipterans, the results were notably different. Several studies showed that a low density of hemipterans benefited more from mutualism than a high density. For example, the difference in the number of survivors between tended and untended treehoppers was highest in small aggregations and decreased significantly as the aggregation size increased [10]. Tending by *F*. *cinerea* significantly improved the growth of small populations of *A*. *varians*, but the benefits from ant tending decreased or disappeared at higher aphid densities [8]. The reason for the decline in benefits may be that the ants were unable to respond to the rapid increase in aphid density. Active recruitment behavior by ant workers did not increase substantially as the hemipteran density increased [32]. Ant workers had a limited requirement for honeydew, which may contribute to the shortage of tending ants [32]. *Lasius niger* would even prey on the aphids *Lachnus tropicalis* and *Myzocallis kuricola* when the aphid density increased [33] because ant workers showed a significant preference for sugars of a different composition [34–35]. The composition of honeydew is influenced by many factors, including the intensity of tending by ants and the quality of the plants [34, 36]. Other studies demonstrated that hemipterans in large aggregations benefited from ant tending more than hemipterans in small aggregations. For example, the time that individual ants of *L*. *niger* were present on scale-infested twigs and the total attendance time of the ants on scale-infested twigs increased significantly as the density of *Ceroplastes rubens* increased [13]. Large aggregations of the membracid *P*. *modesta* benefited more from tending by *F*. *altipetens* than the membracids in small aggregations [9]. Because the amount of honeydew produced is larger with higher hemipteran densities, the larger amounts of honeydew attract a larger number of ants and increased tending levels [37]. Although hemipteran density can influence the intensity of ant-hemipteran mutualisms in opposite directions, the cause of the density dependence may be related to the relative number of tending ants in both cases [38]. Previous studies also reported that hemipterans receive greater benefits when tending levels are high [38–40]. Therefore, the pattern of a density-dependent mutualistic system may be a combined function of the recruitment response of ants mediated by the variation in the size of the hemipteran aggregation [10].

Our study measured whether the benefits to mealybugs from tending by the ghost ant was density-dependent, including mealybug density and parasitoid density. The results showed that the colony growth rate of mealybugs was obviously improved by ant tending, and notably decreased with an increase in the initial mealybug density. However, there was neither an ant tending × mealybug density interaction nor ant tending × parastoid density interaction in our experiment (Table 1, Table 3), which may suggest that the benefits from ghost ant tending were density-independent. Benefits to aphids from ant tending were closely related to the ant tending level (the tending ants per aphid) [8]. Although some studies found a positive correlation between ant foraging activity and the density of hemipterans on plants [3, 41–42], this does not mean that ant tending levels have significantly improved. Our results demonstrated that the positive effect of ant tending on mealybugs did not change significantly as the mealybug density increased, which may suggest that the ghost ant tending level did not vary as the mealybug density increased.

Our result also showed that benefits to mealybugs from ghost ant tending do not depend on parasitoid presence or absence, or on parasitoid density. Whether the primary benefit from ant tending is the protection of hemipteran colonies from natural enemies, the benefit is quite different with various mutualism systems. In some studies, mutualisms between ants and hemipterans primarily focus on protection from natural enemies as the critical means by which the hemipterans benefit [2, 43]. Ant-tended aphids are under intense selective pressures because the ant tending significantly increases the growth rate of aphid colonies when natural enemies were present, and ant tending had a negative influence on the growth and reproduction of the aphids when they were reared free from natural enemies [44–45]. Other studies found that ant tending increased the reproductive output of aphids as a physiological benefit, even though enemies were absent [46–47]. It has been suggested that aphids most likely benefit from ant tending through the stimulation of their feeding rate rather than directly through a decrease in predation rates [6, 48]. Ant-tended treehoppers outperformed untended treehoppers even with predators excluded [10]. Our results showed that no significance was found for the interactions between ant tending × parasitoid (Table 1), which may suggest that mealybugs benefited from ant tending, not only by protection from parasitoids but also when parasitoids were absent. There were also no interactions between ant tending and parasitoid density in our experiments (Table 3). We infer that the effect of increasing parasitoid density is not strong enough to significantly change the level of the protection by ghost ants.

Several studies have shown that the mutualisms between ants and hemipterans are conditional and density-dependent. However, our results indicate that the mutualistic interactions between ghost ants and the invasive mealybug is density-independent. Those results may be the product of the given mealybug density in experiment 1 and 2. In addition, the number of tending ants per mealybug may not have changed significantly as the mealybug density varied. The level of ant tending affected the benefits to hemipterans, which is a widely accepted statement [8–9]. Our results also indicated that there was a positive linear relationship between the ant tending level and the growth rate of the mealybug colony (Fig 5B), which was consistent with previous studies. The results showed that benefits to mealybugs from ant tending fall more at high mealybug densities than at low densities (Fig 5A). The significant difference in the slopes of the two lines may suggest that there was an interaction between mealybug density and ant tending. This result contradicts the results of experiment 1, where no interactions were found. We infer that interactions were only observed at higher mealybug densities, and in experiment 1, we did not examine as wide a range of densities (the highest density was 100 individuals per plant). The pattern of ant-hemipteran benefits may be mediated by various factors, such as the recruitment response of ants, the response and the abundance of natural enemies, and the hemipteran density. Our study adds to a growing number of studies that expound the mechanism of ant-hemipteran mutualisms.

## Supporting Information

S1 Dataset Colony growth rate and percentage parasitism of experiment 1. (XLSX)

S2 Dataset Colony growth rate and percentage parasitism of experiment 2. (XLSX)

S3 Dataset Colony growth rate, percentage parasitism and ant tending level of experiment 3. (XLSX)
